# Applications of Circulating Tumor DNA in a Cohort of Phase I Solid Tumor Patients Treated With Immunotherapy

**DOI:** 10.1093/jncics/pkaa122

**Published:** 2021-01-23

**Authors:** Daniel V Araujo, Ao Wang, Dax Torti, Alberto Leon, Kayla Marsh, Aoife McCarthy, Hal Berman, Anna Spreafico, Aaron R Hansen, Albiruni-Abdul Razak, Philippe L Bedard, Lisa Wang, Eric Plackmann, Helen Chow, Hua Bao, Xue Wu, Trevor J Pugh, Lillian L Siu

**Affiliations:** 1 Division of Medical Oncology and Hematology, Princess Margaret Cancer Centre, University Health Network, Toronto, Ontario, Canada; 2 Geneseeq Technology Inc, Toronto, Ontario, Canada; 3 Ontario Institute for Cancer Research, Toronto, Ontario, Canada; 4 Department of Laboratory Medicine and Pathobiology, University Health Network, Toronto, Ontario, Canada; 5 Department of Biostatistics, Princess Margaret Cancer Centre, University Health Network, Toronto, Ontario, Canada; 6 Department of Medical Biophysics, University of Toronto, Toronto, Ontario, Canada; 7 Princess Margaret Cancer Centre, University Health Network, Toronto, Ontario, Canada

## Abstract

**Background:**

The correlation between blood-based tumor mutation burden (bTMB) and tissue-based tumor mutation burden(tTMB) has not been broadly tested in a multicancer cohort. Here, we assess the correlation between bTMB with tTMB in phase I trial patients treated with immunotherapy. As an exploratory analysis, we evaluated circulating tumor DNA (ctDNA) dynamics in responders.

**Methods:**

Patients treated with immunotherapy at the Princess Margaret phase I trials unit were enrolled. Pretreatment plasma ctDNA and matched normal blood controls were collected. Available archival tissue formalin-fixed paraffin-embedded (FFPE) samples were analyzed. A 425-gene panel was used to sequence both ctDNA and FFPE samples. Samples with TMB within the highest tertile were considered as high TMB.

**Results:**

Thirty-eight patients were accrued from 25 different trials, 86.8% of which involved an anti-PD-1/PD-L1 agent. Thirty patients (78.9%) had detectable mutations in ctDNA, of which the median (range) bTMB was 5 (1-53) mutations per megabase (mut/Mb). Of the 22 patients with available FFPE samples, mutations were detected in 21 (95.4%); the median (range) tTMB was 6 (2-124) mut/Mb. Among the 16 patients with detectable mutations in both FFPE and ctDNA, a statistically significant correlation between bTMB and tTMB was observed (*ρ *= 0.71; *P* = .002). High TMB was not associated with better survival. All 3 responders had a decrease in the variant allele frequency of mutations detected in ctDNA at a second timepoint relative to baseline, indicating a potential early marker of response.

**Conclusions:**

In this small series, bTMB correlated with tTMB. An on-treatment decrease in VAF of mutations detected in ctDNA at baseline was observed in responders. Larger studies to verify our findings are warranted.

Tissue-based tumor mutational burden (tTMB), or the total number of mutations per megabase of coding sequence in a tumor specimen, is a potential predictive marker for response to immunotherapy (1). Increased tTMB has been associated with a higher likelihood of immunotherapy response (2). Determination of tTMB was initially pioneered through whole exome sequencing (WES). However, several recent publications demonstrated that WES tTMB results correlate well with tTMB estimated by large (ie, >300 genes) next generation sequencing (NGS) panels that are more routinely used in clinical practice (3,4). Nonetheless, many other challenges limit the application of tTMB as a selection biomarker for immunotherapy treatment, including logistical aspects such as tissue availability and turnaround time for results; sampling issues due to intratumoral heterogeneity; and technical aspects such as lack of standardization in NGS platforms, cutoffs, and reproducibility (5).

Blood-based TMB (bTMB), the calculation of TMB through analysis of mutations detected in circulating tumor DNA (ctDNA), is an emerging alternative that may overcome some of the barriers associated with tTMB. Pretreatment bTMB correlates well with tTMB in metastatic non-small cell lung cancer (NSCLC) (6,7) and castrate-resistant prostate cancer (8); however, this has not been broadly tested in other cancer types. Beyond bTMB calculation, targeted ctDNA panels may provide prognostic information as well as inform treatment decisions (9). For instance, the identification of driver mutations (eg, *EGFR* T790M in NSCLC) can guide genotype-directed targeted therapy, whereas the identification of resistance mutations (eg, *EGFR* C797S mutation in NSCLC) can prevent futile treatments.

In this pilot study, we analyzed a cohort of advanced solid tumor patients undergoing immunotherapy treatment in an academic phase I clinical trials unit. We hypothesized that bTMB and tTMB would be highly correlated, irrespective of tumor histology, in this heterogeneous pan-patient population. In addition, we postulated that phase I trial patients whose tumors harbor high bTMB would have a favorable clinical outcome in response to immunotherapy treatment. As an exploratory analysis, we evaluated ctDNA dynamics in patients who responded to immunotherapy treatment.

## Methods

### Patients and Samples

From December 2017 to July 2018, patients with metastatic solid tumors seen at the Princess Margaret Cancer Centre phase I trials unit and enrolled in an early phase clinical trial involving investigational immunotherapy treatment were included in this analysis. Investigational immunotherapy treatments such as immune checkpoint inhibitors, vaccines, cytokines, and oncolytic viruses (either as monotherapy, in combination with other immunotherapy agents, or with molecular targeted agents) were included. Combinations involving chemotherapy were not included. Pretreatment and on-treatment whole blood samples were collected in Streck tubes (Cell-Free DNA BCT) and separated into plasma and buffy coat cell fractions in accordance with local standard operating procedures, via an institutional liquid biopsy program (LIBERATE, Liquid Biopsy Evaluation and Repository Development at Princess Margaret, NCT03702309). Circulating tumor ctDNA was extracted from plasma samples collected at pretreatment; midcycle 1 (if feasible); precycle 2, 3, and every other cycle thereafter; and at the end of treatment. Somatic alterations were filtered with matched germline DNA obtained from buffy coat peripheral blood mononuclear cells to remove germline mutations. When available, matched archival formalin-fixed, paraffin-embedded (FFPE) tumor tissues were obtained for genomic characterization. Pretreatment plasma ctDNA, germline DNA, and archival FFPE tumor tissues were assayed. In addition, for selected cases, additional on-treatment samples were assayed to evaluate ctDNA dynamics of patients who responded and did not respond to immunotherapy. This study has ethical approval (18-5815).

### Sample Extraction, Library Preparation, and Sequencing

Whole blood was collected in cell-free (cf) DNA BCT tubes (Streck, La Vista, NE, USA). Plasma and buffy coat were isolated from whole blood after centrifugation at 1900 g for 10 minutes. The plasma layer was further centrifuged at 16 000 g for 10 minutes prior to cfDNA extraction with QIAamp Circulating Nucleic Acid kit (Qiagen, Germantown, MD, USA). Genomic DNA from FFPE tumor tissue and buffy coat was extracted with the AllPrep DNA/RNA FFPE kit and AllPrep DNA/RNA/miRNA Universal kit (Qiagen), respectively. Purified DNA was quantified by Qubit 3.0 using the dsDNA HS Assay kit (ThermoFisher Scientific, Waltham, MA, USA).

A customized panel targeting 425 cancer-relevant genes—GeneseeqPrime—was used for hybridization enrichment (see the Supplementary Appendix, available online). The capture reaction was performed with Dynabeads M-270 (Life Technologies, Carlsbad, CA, USA) and xGen Lockdown hybridization and wash kit (Integrated DNA Technologies, Coralville, IA, USA) according to manufacturers’ protocols. Captured libraries were on-beads polymerase chain reaction (PCR) amplified with Illumina p5 (5’ AAT GAT ACG GCG ACC ACC GA 3’) and p7 primers (5’ CAA GCA GAA GAC GGC ATA CGA GAT 3’) in KAPA HiFi HotStart ReadyMix (KAPA Biosystems, Wilmington, MA, USA), followed by purification using Agencourt AMPure XP beads. Libraries were quantified by quantitative PCR using KAPA Library Quantification kit (KAPA Biosystems). Library fragment size was determined by Bioanalyzer 2100 (Agilent Technologies, Santa Clara, CA, USA). The target-enriched library was then sequenced on HiSeq4000 NGS platforms (Illumina, San Diego, CA, USA) according to the manufacturer’s instructions. The average coverage depth was 5569X (2626-8701X), 1410X (963-2582X), and 354X (285-453X) for plasma, tumor, and normal control samples, respectively. The average Q30 base percentage was 93% for plasma samples, 88% for tumor samples, and 92% normal control samples. Detailed quality control results are presented in Supplementary Table 1 (available online).

### Mutation Calling and TMB Definition

Trimmomatic (10) was used for FASTQ file quality control. Leading and trailing low quality (quality reading below 20) or N bases were removed. Pair-end reads were then aligned to the human reference genome-19 using Burrows-Wheeler Aligner (11) with default parameters. PCR deduplication was performed using Picard V2.9.4 (Broad Institute, Cambridge, MA, USA). Local realignment around indels and base quality score recalibration was performed with the Genome Analysis Toolkit (GATK 3.4.0). Somatic single-nucleotide variants were identified using MuTect (12), and small indels were detected using Scalpel (13). The cutoff for mutation detection was 1% somatic variant allele frequency (VAF) and 5 reads in plasma samples and 2% VAF and 5 reads in FFPE samples. For patients with multiple plasma samples, if a mutation meets the above cutoff in at least 1 sample, the detection cutoff for the same mutation was dropped in other samples to reduce false-negatives. Final list of mutations was annotated using vcf2maf (available on github). TMB was counted by summing all base substitutions and indels in the coding region of targeted genes, including synonymous alterations to reduce sampling noise and excluding known driver mutations because they are overrepresented in the panel, as previously described (14). Samples within the highest mutation-load tertile (top 33.3%) were classified as having high TMB (both for tTMB and bTMB). Discordance in bTMB and tTMB is defined when these values fall into different categories as defined by their respective cutoffs for high vs low TMB (eg, high bTMB and low tTMB, low bTMB and high tTMB).

### Statistical Analysis

Correlations between tTMB and bTMB were calculated using Spearman rank test. Demographics characteristics were summarized in means, medians, and proportions. Associations between categorical variables were examined using the χ^2^ or Fisher exact test. For survival analyses, Kaplan-Meier curves were compared using the log-rank test, and hazard ratios were calculated by Cox proportional hazards model. No formal power calculations were performed for this exploratory analysis. A 2-sided *P* value of less than .05 was considered statistically significant. All statistical analyses were performed in R (v.3.3.2).

## Results

### Patient Characteristics

Among the 40 patients enrolled, 2 were screen failures and did not receive immunotherapy treatment, leaving 38 patients who received at least 1 dose of investigational immunotherapy. This cohort included 25 different phase I studies and 28 tumor types mapped by the OncoTree ontology (15). After grouping, the most frequent tumor sites were colorectal, head and neck squamous cell carcinoma, and breast cancer, all with 5 patients (13.1%) each. The mean age was 59 years old (range = 21-77), and 20 patients (52.6%) were female. Thirty-three (86.8%) patients received an anti-PD-1/anti-PD-L1 immunotherapy treatment, and 31 patients (81.6%) participated in combination trials. Table 1 summarizes the demographic characteristics of the cohort. Baseline plasma sample from all 38 patients and archival FFPE samples from 22 patients (57.8%) underwent NGS targeted sequencing (see the Consort diagram in Supplementary Figure 1, available online).

**Table 1.** Demographic characteristics of enrolled patients (n = 38)

Demographics	No. (%)
Mean age (range), y	59 (21-77)
Female	20 (52.6)
Tumor site	
Colorectal	5 (13.1)
HNSCC	5 (13.1)
Breast	5 (13.1)
Other^a^	23 (60.5)
Treatment	
Involves anti-PD-1/PD-L1 antibodies	33 (86.8)
Combination trial	31 (81.6)
Prior anti-PD-1/PD-L1 antibodies	9 (23.7)
Response rate by RECIST v1.1	
Partial response	3 (7.8)
Stable disease	11 (28.9)
Progressive disease	24 (63.1)

a Others (grouped): esophagus; ovary; neuroendocrine tumor; cholangiocarcinoma; renal cell carcinoma; melanoma; anal squamous cell carcinoma; endometrial; mesothelioma; prostate; pancreas; sarcoma; germ cell tumor; small bowel; small cell lung cancer. HNSCC = head and neck squamous cell carcinoma; RECIST = response evaluation criteria in solid tumors.

Overall, 3 patients (7.8%) achieved partial response (PR), 11 patients (28.9%) achieved stable disease, and 24 patients (63.1%) had progressive disease as best response by response evaluation criteria in solid tumors (RECIST) v1.1. After a median follow-up of 15 months, the median progression-free survival (PFS) for the entire cohort was 1.8 months (95% confidence interval [CI] = 1.76 to 1.92), and the median overall survival was 9.89 months (95% CI = 7.40 to 12.40); the 1-year PFS rate was 13.2% (95% CI = 5.8% to 29.8%), and 1-year OS rate was 39.5% (95% CI = 26.6% to 58.5%).

### Correlation Between bTMB and tTMB and Association With Immunotherapy Efficacy

**Figure 1.**
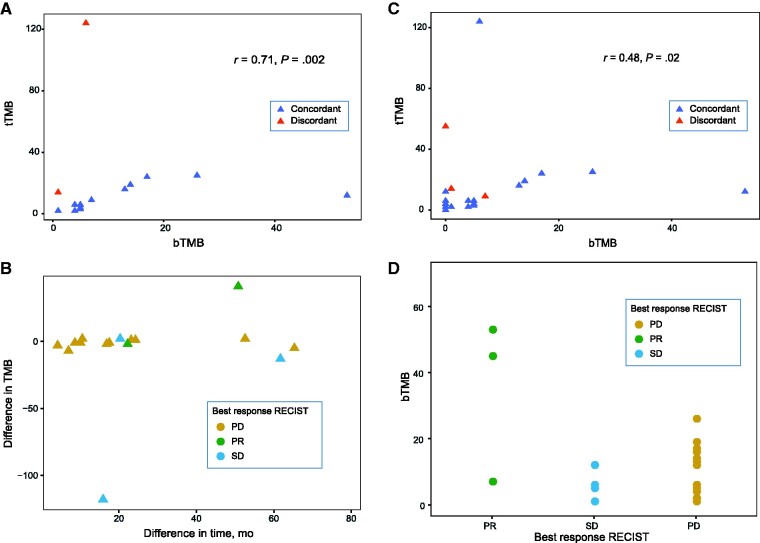
Correlations between bTMB and tTMB; the time interval between samples acquisition and differences in TMB; and the relationship between bTMB and overall response rate. **A)** Spearman correlation of bTMB and tTMB in patients with detectable mutations in both samples (16 pairs). **Dashed lines** = top tertile TMB (12 mut/Mb for both). **B)** Difference between bTMB and tTMB vs time interval between plasma and tissue acquisition. **C)** Spearman correlation of bTMB and tTMB in all patients with matched samples, regardless of mutation detection (22 pairs). **Dashed lines** = top tertile TMB (5 mut/Mb for bTMB and 12 mut/Mb for tTMB). Note that the correlation is higher when mutations are detected in both bTMB and tTMB **(A)**. **D)** Patients who achieved a PR had median TMB higher than SD: 45 mut/Mb vs 1 mut/Mb and PD: 45 mut/Mb vs 5 mut/Mb. bTMB = blood-based tumor mutation burden; PD = progressive disease; PR = partial response; SD = stable disease; tTMB = tissue-based tumor mutation burden.

We detected somatic mutations in 30 of 38 (78.9%) baseline plasma samples and 21 of 22 (95.4%) FFPE samples. The median bTMB was 5 (range = 1-53) mutations per megabase (mut/Mb) and tTMB was 6 (range = 2-124) mut/Mb. Sixteen patients had mutations detected in both plasma and FFPE samples. The Spearman TMB correlation of these 16 pairs was 0.71 (*P* = .002; Figure 1, A). When stratified by top tertile TMB (12 mut/Mb for both sample types, when only these 16 pairs are assessed), 14 of 16 (87.5%) patients have concordant TMB status in both samples. The 2 patients with discordant TMB status had esophageal adenocarcinoma (pt0002) and microsatellite instability-high colorectal cancer (pt0038), and both had higher tTMB than bTMB: 14 vs 1 mut/Mb and 124 vs 6 mut/Mb, respectively, as well as stable disease as best RECIST 1.1 response. Furthermore, both patients were treated with chemotherapy in the interval between FFPE and blood collection acquisition, and pt0038 had 2 distinct *POLE* variants in the FFPE tissue sample, which were not detected in ctDNA. The median time interval between blood and FFPE tissue collection dates was 20 (range = 6.9-64.3) months. The time-interval between blood and FFPE tissue collection was not correlated with the difference in TMB between sample types (Spearman correlation *ρ*  =  0.19, *P* = .48; Figure 1, B). The time interval difference for the 2 patients with discordant TMB results was 60.8 months for pt0002 and 15.6 months for pt0038, respectively.

Next, we performed a sensitivity analysis of the bTMB and tTMB correlation including all patients with matched samples available (22 pairs), regardless of mutation detection. For those subjects with samples without detectable mutations, a TMB of 0 (zero) was considered. (The Spearman correlation was *ρ  = * 0.48, *P* = .02; Figure 1, C). When stratified by top-tertile TMB (5 mut/Mb for bTMB and 12 mut/Mb for tTMB, when 22 pairs are assessed), there were 3 patients with discordant tTMB and bTMB: pt0002 (described previously), pt0016 (a 61-year-old male with metastatic head and neck squamous cell carcinoma with a tTMB of 55 and a bTMB of 0, who had PD as best response) and pt0045 (a 49-year-old woman with metastatic triple-negative breast carcinoma with a tTMB of 9 and a bTMB of 7, who had PR as best response). The time interval between blood and FFPE tissue collection dates for patients pt0016 and pt0045 was 13.8 and 21.9 months, respectively.

Subsequently, we examined whether bTMB or tTMB status was associated with survival or overall response rate. Using a cutoff of 12 mut/Mb, both PFS and OS did not statistically significantly differ between low TMB and high TMB groups, regardless of sample types (Supplementary Figure 2, A-D, available online). However, the 3 PR patients had higher median bTMB compared with both patients with stable disease (45 vs 1) and PD (45 vs 5; Figure 1, D).

### ctDNA Mutational Landscape and Sequential ctDNA Reduction in PR Patients

**Figure 2.**
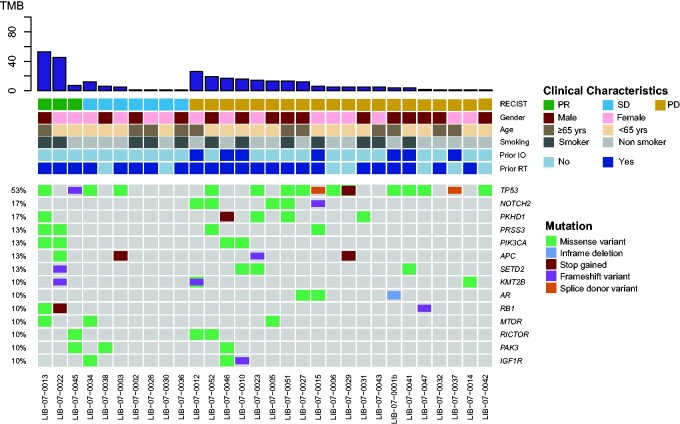
Mutational landscape of baseline plasma samples from 30 patients with detectable mutations. The most frequent mutations identified were *TP53* in 16 (53%), *NOTCH2* and *PKHD1*, in respectively, 5 (17%) and 5 (17%) patients. IO = immunotherapy; PD = progressive disease; PR = partial response; RT = radiotherapy; SD = stable disease; TMB = tumor mutation burden.

Overall, somatic mutations were identified in 156 genes, and 14 genes were encountered in more than 3 patients (Figure 2). The most frequently mutated genes were *TP53* in 16 patients (53.3%), *NOTCH2* in 5 patients (16.6%), and *PKHD1* in 5 patients (16.6%).

**Figure 3.**
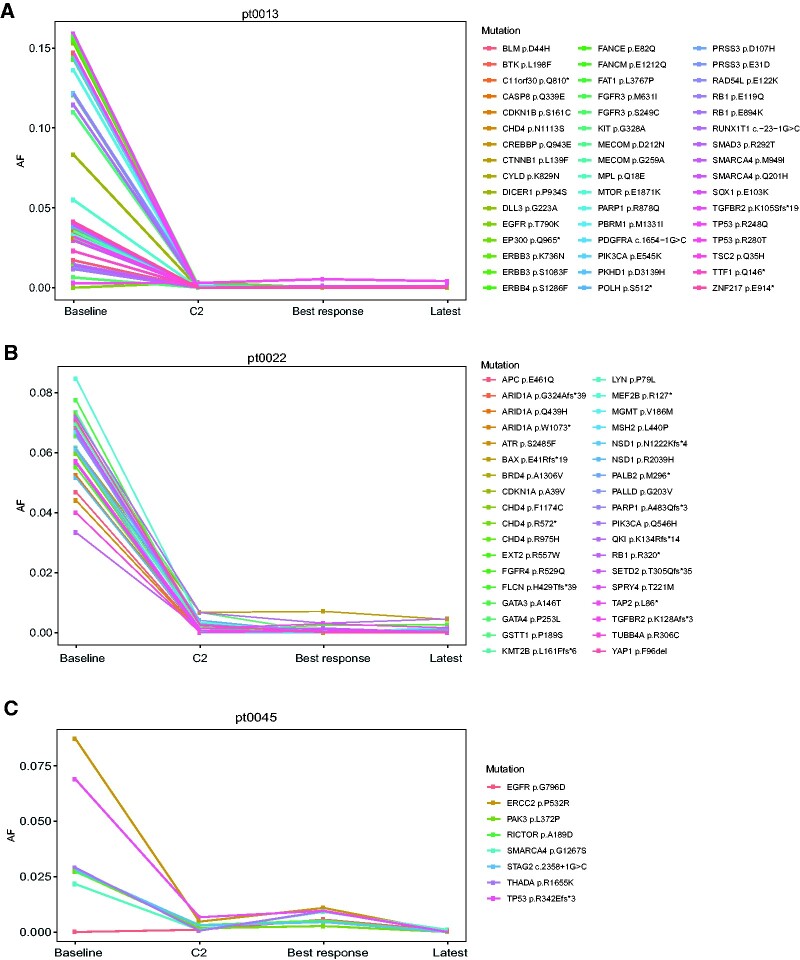
Change in variant allele frequency of circulating tumor DNA mutations encountered at baseline compared with cycle 2. **A)** Anal squamous cell carcinoma; **B)** microsatellite instability-high endometrial carcinoma; **C)** triple-negative breast cancer. AF = allele frequency; C2 = cycle 2.

To monitor the dynamic changes in the VAF of specific mutations detected in plasma samples of the 3 PR patients, we sequenced additional samples collected at the following timepoints: cycle 2 (typically 1 month from baseline), best response, and latest available timepoint. Sequencing results revealed that VAF of mutations detected in the baseline samples were reduced at the second timepoint and stayed minimally detectable through the treatment course (Figure 3, A-C). More specifically, pt0013, a 70-year-old man with metastatic anal squamous cell carcinoma with disease involving lungs, liver, lymph nodes, and peritoneum treated with a combination involving an anti-PD1/PDL1 antibody as first-line regimen, had 48 nonsynonymous mutations present in his baseline plasma sample, 16 of which had VAF of more than 10%. At the second time point, only 9 mutations were detectable and were all less than 1% VAF. Pt0022, a 50-year-old woman with metastatic microsatellite instability-high endometrial carcinoma with locoregional recurrence and lymph node metastasis, treated with a combination involving an anti-PD1/PDL1 agent as second-line regimen, had 36 nonsynonymous mutations present in her baseline plasma sample, with VAF between 1% and 9%. The number of detectable mutations decreased to 25 at the second timepoint, all with less than 1% VAF. Lastly, pt0045, a 49-year-old woman with metastatic triple-negative breast cancer involving lungs and lymph nodes on second-line treatment with a combination involving an anti-PD1/PD-L1 agent, had 7 somatic mutations present in her baseline plasma sample, with VAF between 2% and 9%. In the second sample, all 7 mutations of VAF were reduced to less than 1%.

Next, we sequenced samples of 5 randomly selected patients who had PD as best response (pt0005, a 60-year-old male with metastatic pancreatic neuroendocrine tumor; pt0012, a 76-year-old female with metastatic cutaneous melanoma; pt0015, a 44-year-old female with metastatic triple-negative breast cancer; pt0051, a 65-year-old male with metastatic duodenal adenocarcinoma; and pt0052, a 51-year-old male with metastatic small cell lung cancer). Of these, only pt0005 had an on-treatment decrease of the VAF of ctDNA detectable mutations at baseline (Supplementary Figure 3, A, available online), whereas the remaining 4 patients had either on-treatment stability or increase of the VAF of ctDNA detectable mutations at baseline (Supplementary Figure 3, B-E, available online).

## Discussion

Our results demonstrate a positive correlation between bTMB and tTMB in a multicancer cohort treated with immunotherapy as part of an early phase clinical trial program at the Princess Margaret Cancer Centre. The correlation coefficient (*ρ*  =  0.71; *P* = .002) found in patients with both blood and tissue samples containing detectable mutations in our study is in agreement with the correlation between tTMB and bTMB identified in NSCLC (6,7). Moreover, when using top tertile as a benchmark for defining high TMB (12 mut/Mb for both bTMB and tTMB), only 2 patients had discordant results. Differences between time of blood collection and FFPE acquisition could not explain discrepancies between tTMB and bTMB results in our cohort. However, both patients with discordant results received chemotherapy treatment in the interval between tissue and blood collections, which can potentially account for some of the reduction in bTMB compared with tTMB. A sensitivity analysis including all patients with available samples regardless of mutation detection (22 pairs) demonstrated a positive, but weaker, correlation coefficient (*ρ  = * 0.48; *P* = .02) suggesting that bTMB and tTMB correlation is better when mutations are detected in both sample types. Multiple factors may limit tTMB and bTMB correlation in our study, including heterogeneity of the cohort comprising multiple tumor types with different genomic alterations; differences in the sensitivity of variant detection between FFPE and plasma; variations in tumor cellularity; temporal and spatial heterogeneity of samples; treatment effects; and so forth. Nevertheless, our results suggest that bTMB may be a potential substitute for tTMB for the majority of patients whose tumors harbor detectable mutations in ctDNA.

High TMB (top tertile) assessed by tTMB or bTMB was not associated with better survival outcomes in the current cohort. These findings are similar to data presented by Marabelle et al. of a 755-patient multicancer cohort treated with single agent pembrolizumab (16). High tTMB, defined as 10 mut/Mb or higher in that study, was not associated with improved survival, although patients with high tTMB were more likely to respond to immunotherapy treatment (16). Nonetheless, these findings, akin to our results, may reflect the heterogeneity of pan-cancer patient cohorts and are underpowered to assess the prognostic value of TMB in a tumor-specific context. TMB varies accordingly to cancer type (2), thus different tumor types likely require distinct cutoff values. A universal cutoff value may not be appropriate in pan-cancer analyses. Samstein et al., analyzing genomic data of 1662 patients who underwent NGS targeted sequencing (MSK-IMPACT, with 468 genes) and received an immunotherapy-based treatment, found that within the same histology, the higher the tTMB, the higher the likelihood of better survival outcomes (17). In this MSK-IMPACT study, the highest mutation-load quintile (top 20%) for each tumor type was selected as the cutoff to define high TMB, and the authors found that different tumors yield different cutoffs (eg, breast 5.9 mut/Mb and melanoma 30.7 mut/Mb). Because of our limited sample size and few patients with objective responses (3 PRs), we have not performed analysis per histology. However, pt0045 in our cohort, a patient with metastatic triple-negative breast cancer whose blood sample had a bTMB of 7 mut/Mb—typically classified as low but considered high in the context of breast cancer—responded to immunotherapy treatment, supporting the histology-based TMB cutoff hypothesis. Nevertheless, further research is needed to translate the application of TMB into clinical practice and confirm its clinical utility, as this biomarker has been the subject of pronounced scrutiny (3,5). Some of the challenges lie in the lack of TMB definitions, standardization of assays, variant filtering methods, sequencing technologies, cutoffs, and reporting of TMB results (18). Working groups have been established in an attempt to harmonize some of these aspects (19).

Mutations in ctDNA were observed in 79% of patients assayed with the GeneseeqPrime panel in this cohort, which is comparable to the sensitivity of other commercially available ctDNA panels testing a similar number of genes (6,20,21). The ctDNA VAFs of all 3 responders had decreased in the blood sample collected at cycle 2 relative to baseline sample, whereas 4 of 5 patients with PD had either on-treatment stability or increase in ctDNA VAF of detectable mutations. One patient, with discordant bTMB vs tTMB results with PD as best response, had on-treatment decrease in ctDNA VAF of detectable mutations. In contrast to the 3 patients with PR as best response whose on-treatment ctDNA mutations had VAF of less than 1%, most of the PD patients’ on-treatment ctDNA mutations had VAF of greater than 1%. There is a general association between cfDNA mutation VAF clearance and treatment responses in different tumor types (22,23). For instance, in a cohort of patients with metastatic *PIK3CA*-mutated breast cancer treated with fulvestrant and palbociclib, a posttreatment decrease in plasma ctDNA *PIK3CA* levels below the median at cycle 1, day 15, compared with baseline levels was associated with improved PFS (23). Nevertheless, many factors determine the ability of a targeted panel to detect ctDNA mutations over time. Foremost, there is usually a trade-off between panel size and reading depth. For instance, applicability of WES in ctDNA is currently limited because of shallow coverage and thus may not be able to track mutations that exist at very low levels. Conversely, despite having an adequate reading depth, small-sized panels are applicable mainly in scenarios where the genomic regions of interest are known or anticipated (eg, *BRAF* mutation in *BRAF* mutant melanomas) but are not comprehensive enough to be useful in an unselected scenario. A potential solution is the emergence of bespoke small-sized ctDNA panels with great read depths targeting mutations identified from WES (from pretreatment tumor samples), which are highly sensitive for ctDNA detection. This type of bespoke ctDNA analysis was recently applied by our group in a multicancer cohort treated with single agent pembrolizumab (14,24), dynamic changes in the VAFs of patient-specific ctDNA samples collected on-treatment compared with baseline predicted for treatment response, PFS, and OS. Our current data suggest that a large ctDNA panel may also be able to provide early response markers of immunotherapy treatment effectiveness. Although the coverage depth of large ctDNA panels is inferior to a bespoke approach, large panels have the advantages of being readily available, potentially more scalable, and not reliant on access to tumor WES results. In addition, large ctDNA panels have the potential advantage of capturing molecular progression caused by emergence of new mutations not previously detected at baseline (25).

This study has several limitations, and results should be interpreted with caution. First, our cohort is small and includes patients with multiple tumor types and distinct genomic alterations. Second, patients receiving treatment across 21 different trials were included; nevertheless, 87% of patients received an anti-PD1/PD-L1 checkpoint inhibitor as part of their treatment. Third, 42% of patients had no available archival tissue, preventing a more definitive analysis of tTMB/bTMB correlation. Lastly, our small sample size prohibited multivariable adjustments.

In conclusion, our work demonstrates that when mutations are detected in both tissue and blood samples, tTMB and bTMB are highly correlated in a diverse multitumor phase I cancer clinical trials patient cohort. Blood-based TMB may be an alternative to tTMB in patients with advanced solid tumors with detectable mutations in ctDNA. An exploratory analysis suggests that early decrease in VAF of ctDNA mutations may be a marker of immunotherapy response. Larger studies addressing these hypotheses are warranted.

## Funding

This work was supported by the BMO Chair in Precision Genomics and Geneseeq Technology Inc.

## Notes

**Role of the funder:** The funder had no role in the design of the study; the collection, analysis, and interpretation of the data; the writing of the manuscript; and the decision to submit the manuscript for publication.

**Disclosures:** DVA has received honoraria from GSK; AW, HB, and XW are employees of Geneseeq Inc; DT, LW, KM, AL, AM, HB, EP, and HC have no conflicts to report; AS has the following financial relationships to disclose (2020): Consulting for: Bristol-Myers Squibb, Merck, Novartis, Oncorus; Research funding: Alkermers, Array, BioPharma, Astrazeneca/MedImmune, Bayer, Bristol-Myers Squibb, Janseen Oncology, Merck, Northern Biologics, Novartis, Replimune, Roche, Surface Oncology, Symphogen. ARH has the following financial relationships to disclose (2020): Honoraria: AstraZeneca/MedImmune, Bristol-Myers Squibb; GlaxoSmithKline/Novartis, Merck, Pfizer; Consulting role: Boehringer Ingelheim, Boston Biomedical, Bristol-Myers Squibb, Genentech/Roche, GlaxoSmithKline, Merck, Novartis; Research funding: Boehringer Ingelheim, Bristol-Myers Squibb, GlaxoSmithKline, Janseen, Karyopharm Therapeutics, Merck, Novartis, Roche/Genentech. PLB has the following financial relationships to disclose: Research funding (clinical trials for institution): AstraZeneca, Bristol-Myers Squibb, Genentech/Roche, GlaxoSmithKline, Immunomedics, Lilly, Merck, Mersana, Nektar, Novartis, PTC Tehrapeutics, Sanofi, Seattle Genetics, Servier, SignalChem. TJP provides consultation for Merck, Chrysalis Biomedical Advisors, and the Canadian Pension Plan Investment-Board and receives research support from Roche; AAR has the following financial relationships to disclose: Honoraria: Boehringer Ingelheim; Consulting role: Boehringer Ingelheim, Lilly and Merck; Research funding: Abbvie; Adaptimmune, Angen, Blueprint Medicines, Boehringer Ingelheim, Boston Biomedical, Bristol-Myers Squibb, CASI Pharmaceuticals, Deciphera, GlaxoSmithKline, Karyopharm Therapeutics, Lilly, MedImmune, Merck, Novartis, Pfizer, Roche/Genentech. LLS has the following financial relationships to disclose (2020): Consultant for: Merck (compensated), Pfizer (compensated), Celgene (compensated), AstraZeneca/Medimmune (compensated), Morphosys (compensated), Roche (compensated), GeneSeeq (compensated), Loxo (compensated), Oncorus (compensated), Symphogen (compensated), Seattle Genetics (compensated), GSK (compensated), Voronoi (compensated), Treadwell Therapeutics (compensated), Arvinas (compensated), Tessa (compensated), Navire (compensated), Relay (compensated), Rubius (compensated); Grant/Research support from (Clinical Trials for institution): Novartis, Bristol-Myers Squibb, Pfizer, Boerhinger-Ingelheim, GlaxoSmithKline, Roche/Genentech, Karyopharm, AstraZeneca/Medimmune, Merck, Celgene, Astellas, Bayer, Abbvie, Amgen, Symphogen, Intensity Therapeutics, Mirati, Shattucks, Avid; Stockholder in: Agios (spouse); Leadership in: Treadwell therapeutics (spouse = cofounder).

**Author contributions:** DVA and LLS conceived the study. AW performed data analysis. DVA reviewed clinical data and performed data analysis. AS, PLB, ARH, AAR, and LLS accrued patients and supervised biospecimen collection. TJP and DT supervised biospecimen sample processing by AL and KM. HC and EP provided logistical support to the project. AM and HB performed the pathology review of samples. AW, HB, and XW conducted the sequencing assays and provided technical expertise and scientific feedback. AW and LW conducted the statistical analysis. DVA, AW, XW, and LLS wrote the manuscript. All authors discussed the results and implications and commented on the manuscript at all stages.

**Acknowledgements:** We would like to thank all patients and their families for participating in this study.

## Data Availability

The data underlying this article cannot be shared publicly due to confidentiality agreements with sponsors of ongoing/completed Phase I studies from Princess Margaret Cancer Centre. This is a deidentified secondary analysis. The data may be shared upon reasonable request to the corresponding author.

## Supplementary Material

pkaa122_Supplementary_Data
