# Supplementary material for: Applications of Circulating Tumor DNA in a Cohort of Phase I Solid Tumor Patients Treated With Immunotherapy
Source: JNCI Cancer Spectr. 2021 Jan 23;5(3):pkaa122. doi: 10.1093/jncics/pkaa122 (PMC8152803; doi:10.1093/jncics/pkaa122)
Supplement: pkaa122_Supplementary_Data [file pkaa122_supplementary_data.zip › CS20-0080R2 Araujo supp mat_AB.pdf]

SUPPLEMENTARY MATERIALS

Supplementary Appendix—*Available for separate download*.

Supplementary Table 1—*Available for separate download*.

Supplementary Figure 1 – Consort diagram.

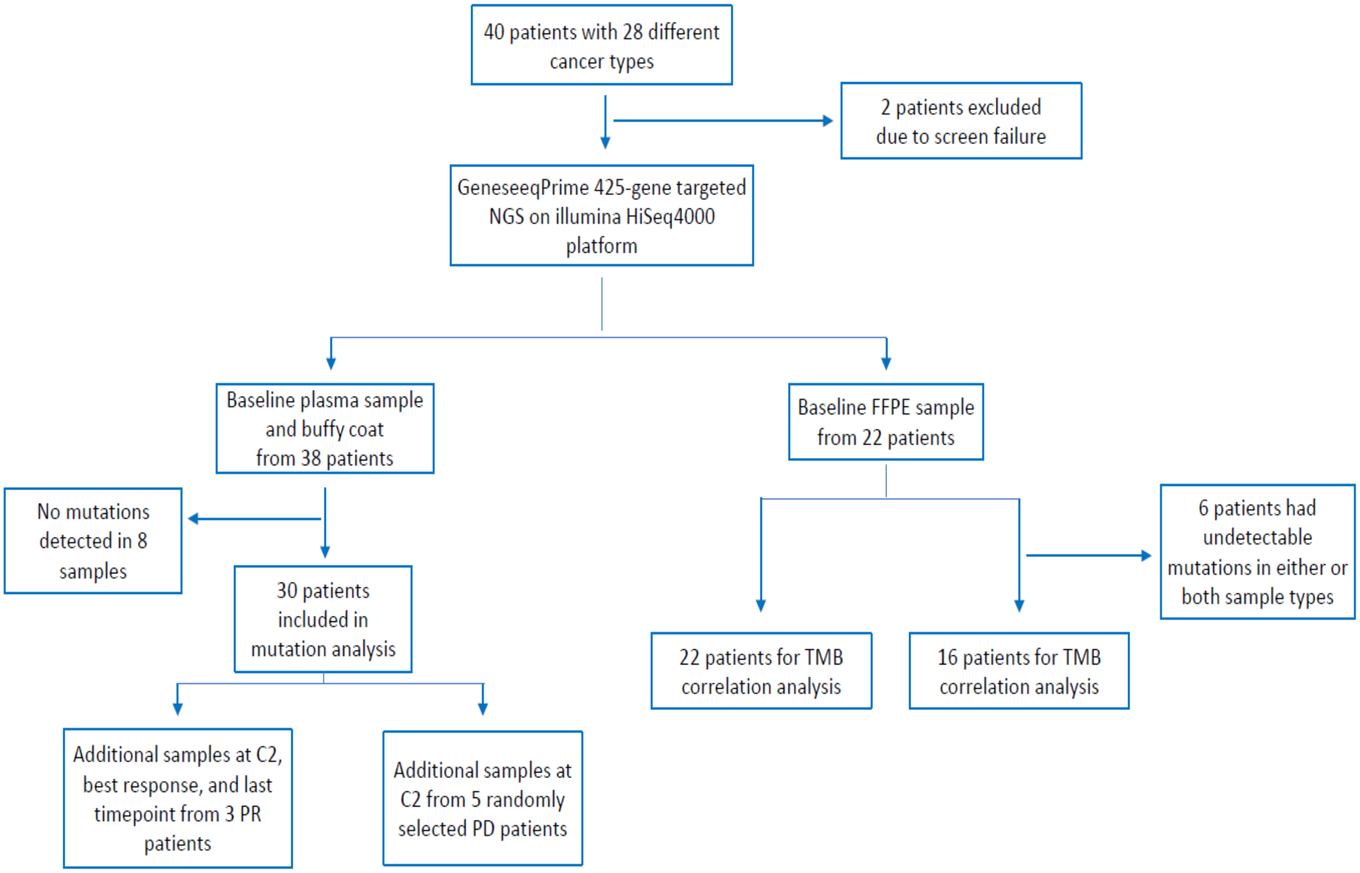

Supplementary Figure 2. Using a cut-off of 12 (higher tertile) for both bTMB and tTMB, there was no association with either PFS or OS in both bTMB (A and B) or tTMB (C and D).

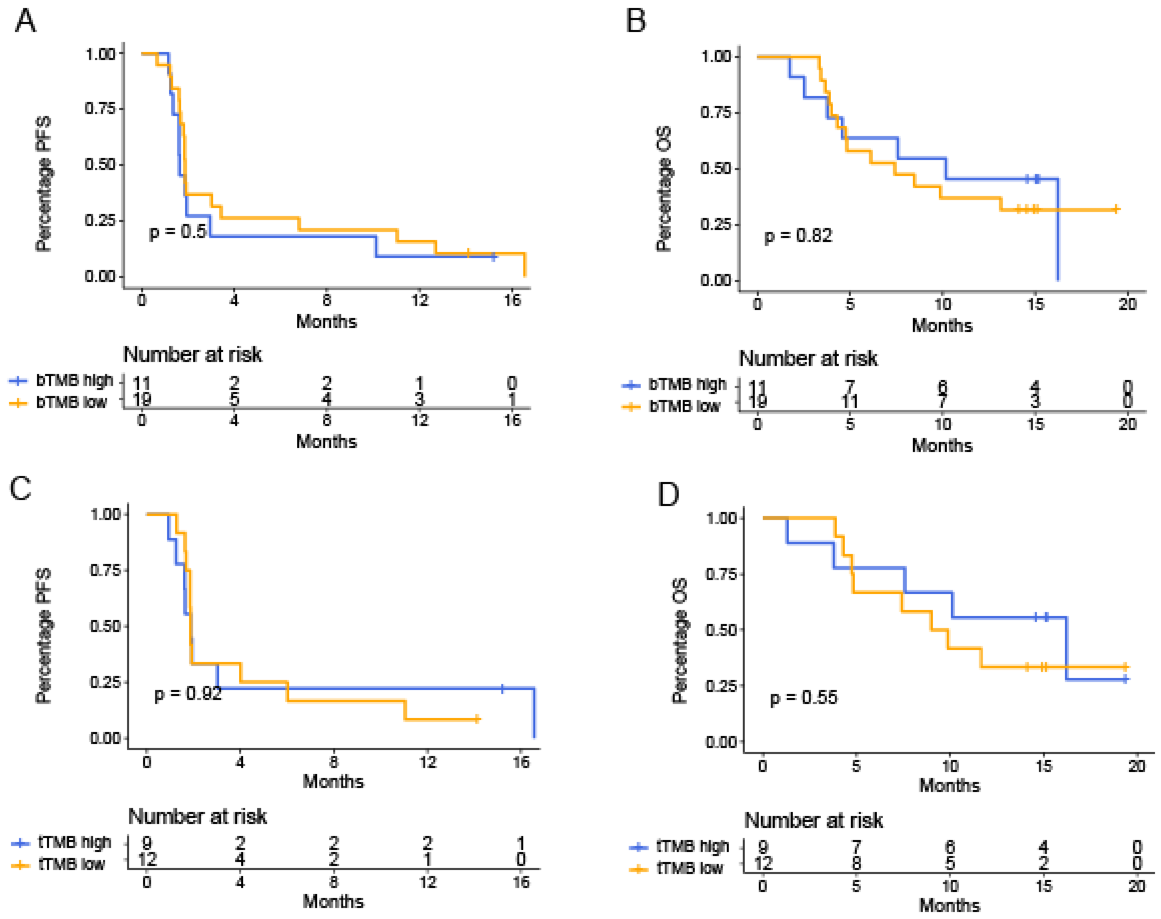

Supplementary Figure 3 - A-E. On-treatment changes in VAF of ctDNA encountered at baseline of 5 randomly selected patients who had PD as best response. A. Pancreatic neuroendocrine tumor. B. Cutaneous melanoma. C. Triple negative breast cancer. D. Duodenal adenocarcinoma. E. Small cell lung cancer.

A)

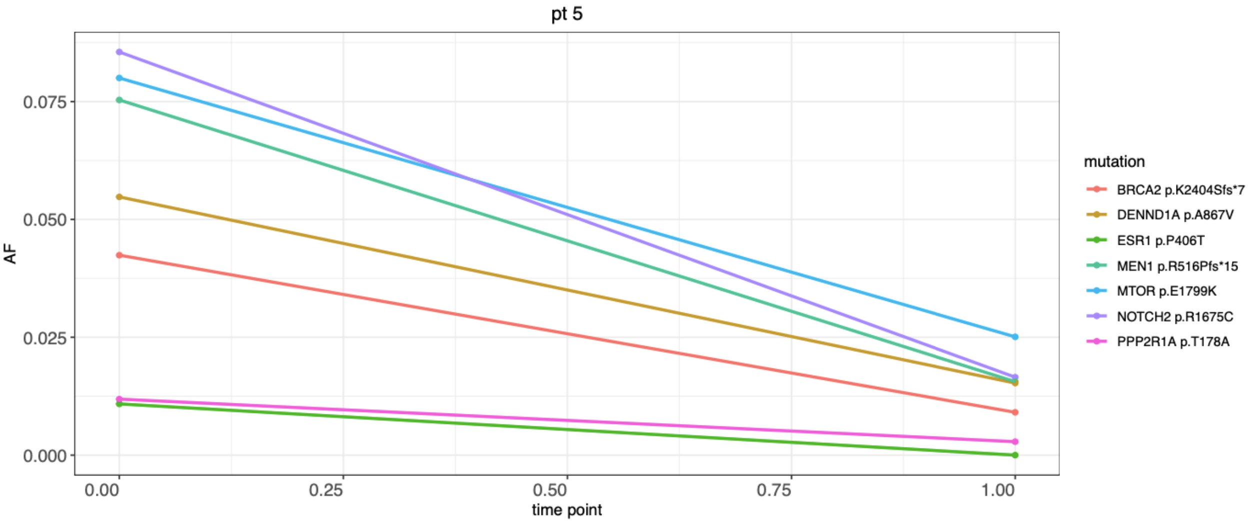

B)

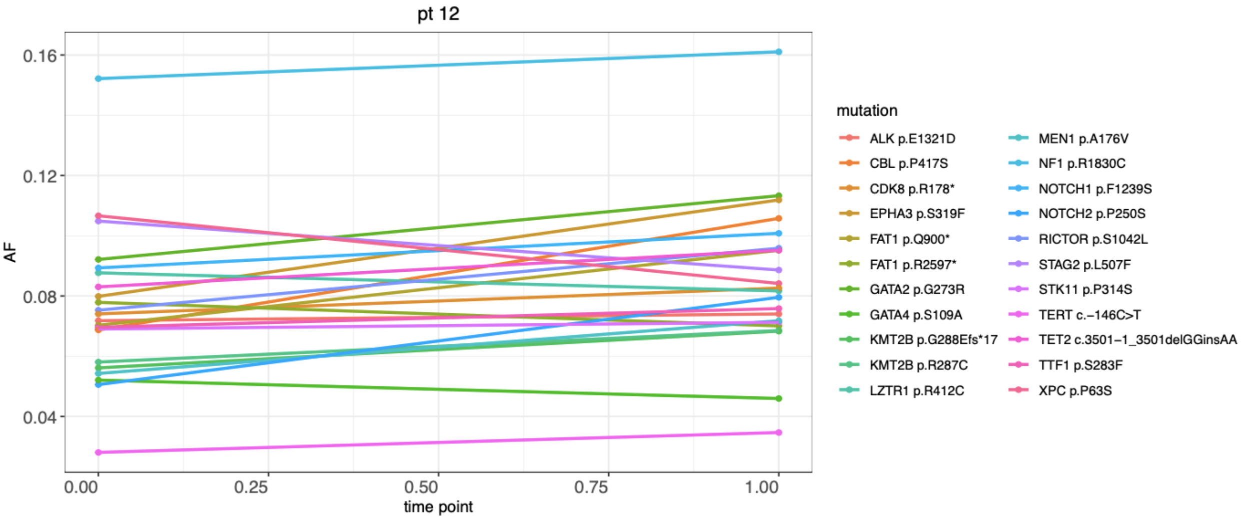

C)

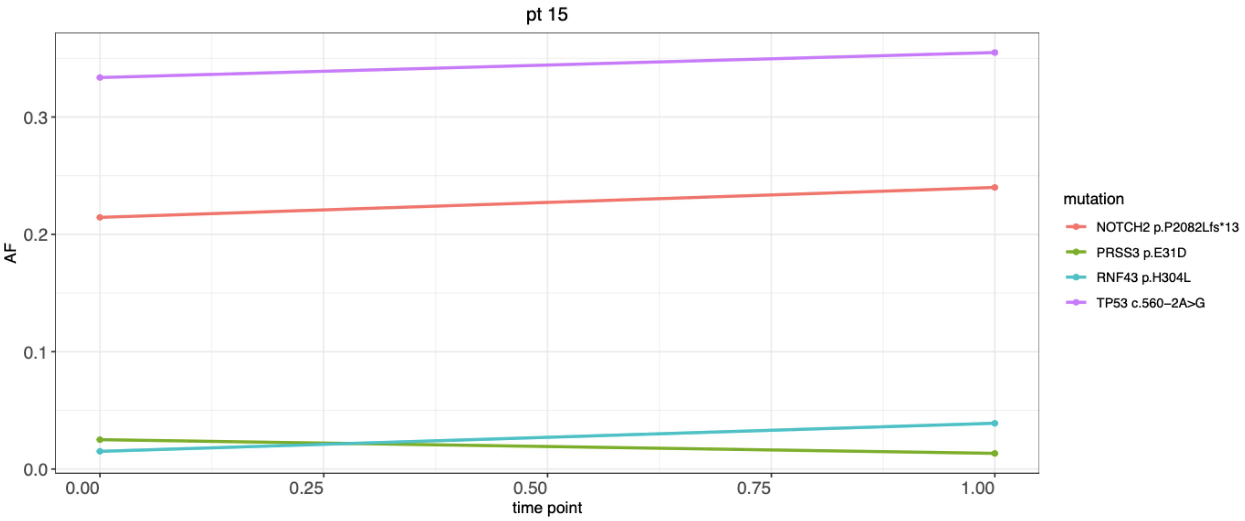
pt 15
AF
0.0
0.1
0.2
0.3
0.00
0.25
0.50
0.75
1.00
time point
mutation
NOTCH2 p.P2082Lfs*13
PRSS3 p.E31D
RNF43 p.H304L
TP53 c.560−2A>G

D)

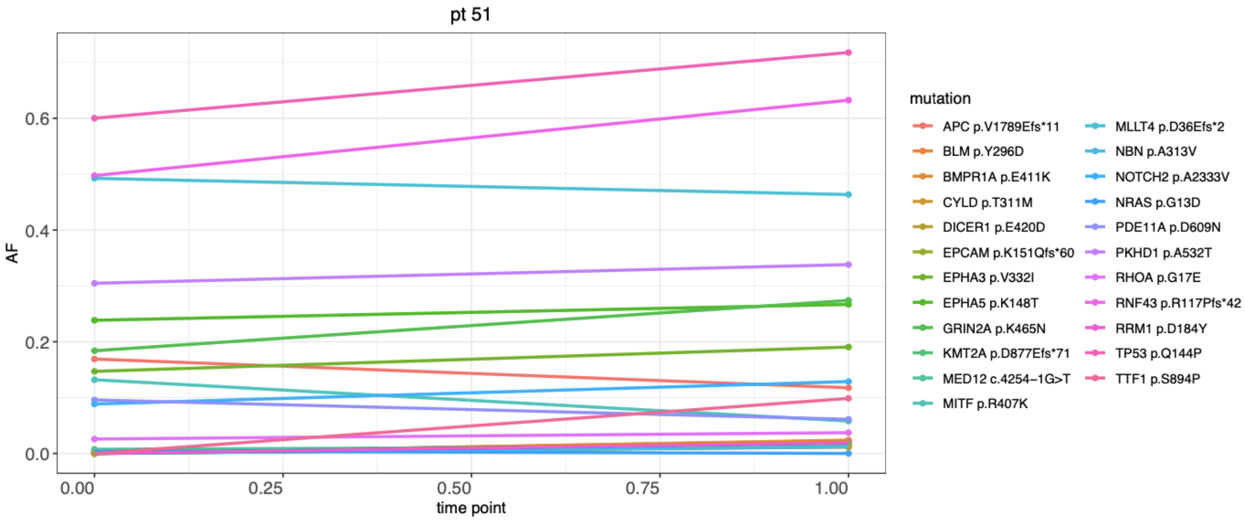
pt 51
AF
0.0
0.2
0.4
0.6
0.00
0.25
0.50
0.75
1.00
time point
mutation
APC p.V1789Efs*11
BLM p.Y296D
BMPR1A p.E411K
CYLD p.T311M
DICER1 p.E420D
EPCAM p.K151Qfs*60
EPHA3 p.V332I
EPHA5 p.K148T
GRIN2A p.K465N
KMT2A p.D877Efs*71
MED12 c.4254−1G>T
MITF p.R407K
MLLT4 p.D36Efs*2
NBN p.A313V
NOTCH2 p.A2333V
NRAS p.G13D
PDE11A p.D609N
PKHD1 p.A532T
RHOA p.G17E
RNF43 p.R117Pfs*42
RRM1 p.D184Y
TP53 p.Q144P
TTF1 p.S894P

E)

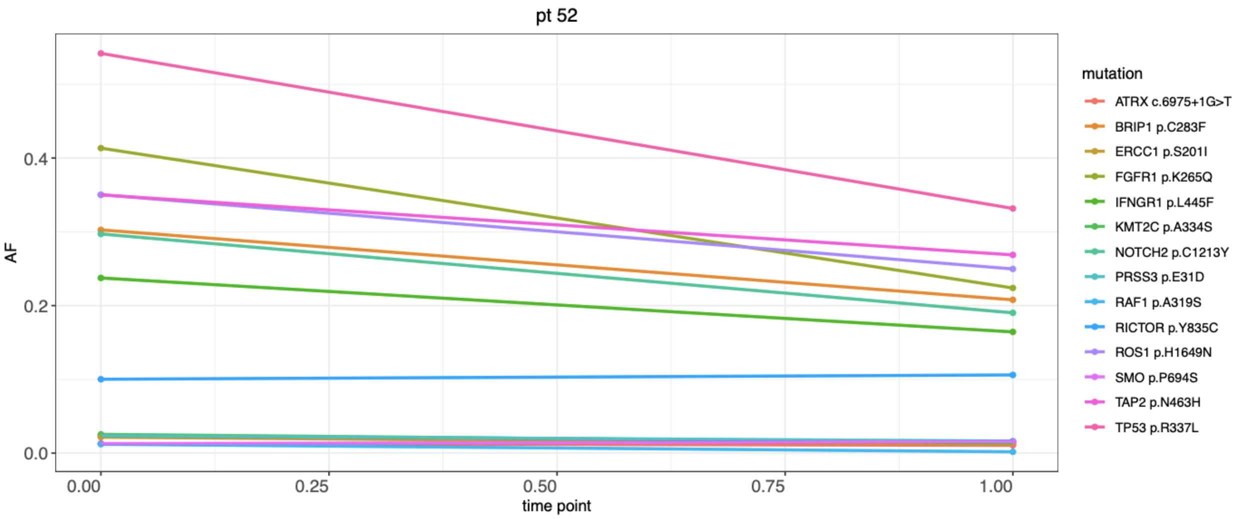
pt 52
AF
0.0
0.2
0.4
0.00
0.25
0.50
0.75
1.00
time point
mutation
ATRX c.6975+1G>T
BRIP1 p.C283F
ERCC1 p.S201I
FGFR1 p.K265Q
IFNGR1 p.L445F
KMT2C p.A334S
NOTCH2 p.C1213Y
PRSS3 p.E31D
RAF1 p.A319S
RICTOR p.Y835C
ROS1 p.H1649N
SMO p.P694S
TAP2 p.N463H
TP53 p.R337L
